# Inhibition of cell invasion and migration by targeting matrix metalloproteinase-9 expression via sirtuin 6 silencing in human breast cancer cells

**DOI:** 10.1038/s41598-022-16405-x

**Published:** 2022-07-15

**Authors:** On-Yu Hong, Hye-Yeon Jang, Young-Rae Lee, Sung Hoo Jung, Hyun Jo Youn, Jong-Suk Kim

**Affiliations:** 1 Department of Biochemistry, Institute for Medical Sciences, Jeonbuk National University Medical School, 20 Geonji-ro, Deokjin, Jeonju, Jeollabuk 54907 Republic of Korea; 2 Department of Oral Biochemistry, and Institute of Biomaterials, Implant, School of Dentistry, Wonkwang University, Iksan, Jeollabuk 54538 Republic of Korea; 3 Department of Surgery, Research Institute of Clinical Medicine of Jeonbuk National University and Biomedical Research Institute of Jeonbuk National University Hospital, 20 Geonji-ro, Deokjin, Jeonju, Jeollabuk 54907 Republic of Korea

**Keywords:** Biochemistry, Cancer, Molecular biology

## Abstract

Sirtuin 6 (SIRT6) regulation is involved in carcinogenesis. However, its role in breast cancer (BC) metastasis remains unclear. We investigated the effects of SIRT6 on protein kinase C activator- and cytokine-mediated cancer cell invasion and migration in MCF-7 and MDA-MB-231 cells and the association between SIRT6 and matrix metalloproteinase-9 (MMP-9) expression. To assess MMP-9 and SIRT6 expression in patients, protein levels in BC tissues were analyzed. MCF-7 and MDA-MB-231 cell viability was analyzed using MTT assays. SIRT6 was silenced in both cell lines and protein secretion, expression, and mRNA levels were analyzed. Transcription factor DNA activity was investigated using luciferase assays. Matrigel invasion assays were used to assess the effects of SIRT6 in both cell lines. SIRT6 and MMP-9 expression in cancer tissues was significantly higher than in paired normal breast tissues. 12-O-tetradecanoylphorbol-13-acetate (TPA) or tumor necrosis factor-α (TNF-α) increased MMP-9 expression and cell invasion and migration, but SIRT6 knockdown abolished these effects. SIRT6 overexpression additively increased TPA- and TNF-α-induced MMP-9 expression. SIRT6 knockdown suppressed the mitogen-activated protein kinase (MAPK) signaling pathway and thus TPA- and TNF-α-induced MMP-9 expression. SIRT6 silencing suppressed TPA- and TNF-α-induced nuclear factor-κB (NF-κB) and activator protein-1 (AP-1) expressions in both cell lines, and treatment with MAPK, NF-κB, and AP-1 inhibitors reduced MMP-9 expression. The anti-invasive effects of SIRT6 in BC cells might be mediated by suppression of MAPK phosphorylation and reduction in NF-κB and AP-1 DNA activities, leading to MMP-9 downregulation, suggesting that SIRT6 modulation has the potential to target BC metastasis.

## Introduction

Breast cancer is the most common type of cancer and the major cause of cancer-related death in women^1^, and most deaths from breast cancer are attributed to tumor metastasis. Therefore, the control of cell invasiveness represents a crucial therapeutic strategy for breast cancer, and the expansion of valid anti-invasive agents offers a potentially effective means of improving treatment^2^. Cancer cell invasion involves biological changes, such as the loosening of tissue structures and extracellular matrix (ECM) proteolysis^3^. ECM degradation is a hallmark of cancer metastasis and is the effect of excessive secretion of proteolytic enzymes, such as matrix metalloproteinases (MMPs)^4^. MMP-9 is a key enzyme that plays a major role in enhancing tumor cell invasion and migration^5^. Therefore, MMP-9 expression has been extensively used as a marker for breast cancer metastasis^6^. The expression of MMP-9 can be upregulated by growth factors, chemokines, cytokines, and protein kinase C (PKC) activators, such as tumor necrosis factor-α (TNF-α) and 12-O-tetradecanoylphorbol-13-acetate (TPA). There are potent tumor promoters that can induce MMP-9 expression through various intracellular signaling pathways^7^.

MMP-9 is regulated at the transcriptional level by activator protein-1 (AP-1) or nuclear factor-κB (NF-κB)^8^. In addition, the induction of mitogen-activated protein kinase (MAPK) and phosphoinositide-3-kinase (PI3K) signaling is involved in MMP-9 expression^9^. PKC is also a well-known key factor in signal transduction. PKC isoforms are important for tumor promotion and activation of the MMP-9-related signaling pathway^10^. It is important to understand the molecular mechanisms underlying MMP-9 expression in order to identify novel targets and prevent metastasis.

Sirtuin 6 (SIRT6) is a member of the sirtuin family, NAD+-dependent histone deacetylases. SIRT6 is associated in aging-related diseases and reported that SIRT6 deletion showed an aging-like phenotype^11^ and is implicated in cellular signaling pathways such as lifespan, metabolism, DNA damage repair, cell cycle regulation, and apoptosis^12^. SIRT6 has been reported to play an oncogenic role in many human cancers by accelerating the cell cycle process and tumor growth^13^, reducing apoptosis^14^, and increasing cancer cell invasiveness^15^, thus promoting tumor progression. However, some reports SIRT6 as a tumor suppressor that regulates tumor formation and maintenance of cancer^16^. Nevertheless, SIRT6 in our study was investigated as a factor causing cancer metastasis. Although the involvement of SIRT6 in tumorigenesis has been frequently reported, only a small number of studies have addressed its role in breast cancer metastasis.

This study investigated the effect of SIRT6 on the invasive behavior of two breast cancer cell lines, MCF-7 and MDA-MB-231, as well as the role played by MMP-9.

## Materials and methods

### Reagents

TPA (20 nM) and β-actin antibodies were purchased from Sigma-Aldrich (St. Louis, MO, USA). Recombinant human tumor necrosis factor-α (TNF-α; 10 ng/mL) was purchased from R&D Systems (Minneapolis, MN, USA). Inhibitors of AP-1 (SR 11302) and NF-κB (Bay 11-7092) were purchased from Santa Cruz Biotechnology (Santa Cruz, CA, USA). The MAPK inhibitors SB203580 (p38 inhibitor), SP600125 (JNK inhibitor), and PD98059 (ERK inhibitor) were purchased from Merck Millipore (Billerica, MA, USA). Rabbit antibodies against SIRT6, phosphorylated (p-)-c-Jun, c-Jun, p-c-Fos, c-Fos, p-IKKα/β, IKKα, IKKβ, p-IκBα, SAPK/JNK, p-SAPK/JNK, p38 MAPK, p-p38 MAPK, p44/42 MAPK (Erk1/2), p-p44/42 MAPK (Erk1/2), and PKCδ were purchased from Cell Signaling Technology (Beverly, MA, USA). Rabbit antibodies against NF-κB p65, NF-κB p105/p50, MMP-9, PKCα, and PKCβ were purchased from Abcam (Cambridge, UK). Mouse antibodies against IκBα were purchased from Cell Signaling Technology. Mouse anti-PCNA antibodies were obtained from Santa Cruz Biotechnology. The secondary antibodies anti-rabbit IgG, HRP-linked antibody, anti-mouse IgG, and HRP-linked antibody were purchased from Cell Signaling Technology. We used both the primary and second antibodies at a 1000:1 dilution (in 5% skim milk/1X TBS).

### Cell culture

MCF-7 and MDA-MB-231 human breast cancer cell lines were obtained from the American Type Culture Collection (ATCC; Manassas, VA, USA). Cells were cultured in a 5% CO_2_ incubator at 37 °C in high-glucose Dulbecco's modified Eagle's medium (DMEM) supplemented with 1% antibiotics (10,000 units/mL penicillin and 10,000 μg/mL streptomycin) and 10% fetal bovine serum (FBS). High-glucose DMEM, phosphate-buffered saline (PBS), and fetal bovine serum (FBS) were obtained from Gibco (Thermo Fisher Scientific, Waltham, MA, USA).

### MTT assay

MCF-7 and MDA-MB-231 cells were seeded into a 96-well plate and incubated at 37 °C for 24 h to allow attachment. Cells were either untreated or treated with TPA or TNF-α at 37 °C for 24 h and then washed with phosphate-buffered saline (PBS; Gibco; Thermo Fisher Scientific). MTT assays were then performed using 0.5 mg/mL MTT (Sigma-Aldrich; Merck KGaA). Following the addition of MTT, the cells were incubated at 37 °C for 30 min. Dimethyl sulfoxide was added to dissolve the formed formazan crystals, and the optical absorbance at 570 nm was determined using a microplate reader (Bio-Rad Laboratories, CA, USA).

### Transfection with small interfering RNA (siRNA)

Duplexes of small interfering RNA (siRNA) targeting human SIRT6 mRNA (target sequences are as follows: si-SIRT6: CAGCUUAAACAGGAGUGAA for sense and UUCACUCCUGUUUAAGCUG for antisense). SIRT6-specific siRNA and negative control siRNA were obtained from BIONEER (Daejeon, Korea), Opti-MEM medium was obtained from Gibco, and Lipofectamine RNAimax was purchased from Invitrogen (Carlsbad, CA, USA). The cells were transfected according to the manufacturer’s instructions (Invitrogen). Cells were harvested and re-suspended in Opti-MEM medium with Lipofectamine RNAimax, mixed with 50 pmol siRNA, and transfected for 12 h. *SIRT6* knockdown was confirmed by western blotting and real-time polymerase chain reaction (RT-qPCR) assays.

### Virus infection

Cells were placed in culture plates and infected with adenoviruses for 24 h in DMEM. The concentration of adeno-SIRT6 virus (multiplicity of infection, 300–500) used (as determined in a preliminary study) was equal to that of adeno-LacZ virus, which allowed infection of each cell line without toxic effects in a pre-experiment. Adenovirus expressing SIRT6 (provided by Professor Park Byeong-hyeon, Jeonbuk National University, Jeonju, South Korea), Production of AdSirt6 was described to in Park's paper^17^.

### Isolation of nuclear and cytoplasmic extracts

Transfected cells were treated with TPA or TNF-α for 3 h, washed with PBS, and pelleted by centrifugation. Nuclear and cytoplasmic extracts were prepared using NE-PER cytoplasmic and nuclear extraction reagents (Thermo Fisher Scientific). Nuclear and cytoplasmic protein fractions were obtained according to the manufacturer’s instructions (Thermo Fisher Scientific).

### Membrane fraction

Transfected cells were treated with TPA or TNF-α for 40 min, washed with PBS, and pelleted by centifugation. Cytoplasmic and membrane protein extracts were prepared using the Mem-PER Plus Membrane Protein Extraction Kit (Thermo Fisher Scientific), according to the manufacturer’s instructions (Thermo Fisher Scientific).

### Western blotting analysis

Proteins were extracted using mammalian protein extraction reagent (M-PER; Pierce Biotechnology) in the presence of a proteinase inhibitor. Protein concentration was determined using a Bio-Rad assay (Bio-Rad Laboratories, Inc.). Cell lysates (10 μg protein) were resolved using 10% SDS-PAGE and transferred to Hybond™-polyvinylidene fluoride membranes (GE Healthcare Life Sciences, Buckinghamshire, UK), which were then blocked for 2 h at 4 °C with skim milk or bovine serum albumin (5% in 1X TBS; purchased from MP Biomedicals, LLC, OH, USA), incubated overnight at 4 °C with primary antibody (1:1000; in 5% skim milk/1 × TBS), and then incubated with secondary antibody, HRP-conjugated IgG (1:1000 dilution in 1 × TBS) for 1 h at 4 °C. Immunoreactive signals were visualized using an electrochemiluminescent HRP substrate peroxide solution and luminol reagent (Merck Millipore). Protein levels were measured using an imaging system (Las-4000; FujiFilm Corporation, Tokyo, Japan) and image analyzer software (Multi-Gauge v.3.0; FujiFilm Corporation).Because the blots were cut prior to hybridization with antibodies during blotting, it was difficult to provide images showing full-length membranes.

### Zymography assay

Conditioned media were collected, mixed with sample buffer (non-reducing loading buffer), and separated by PAGE with gelatin (0.1%). The gel was washed for 30 min with Triton X-100 solution (2.5%) at 37 °C and incubated for 16 h in developing buffer (or digestion buffer; composition: 5 mM CaCl_2_, 0.02% Brij, and 50 mM Tris–HCl, pH 7.5) at 37 °C. The gel was stained for 30 min in 0.25% Coomassie Brilliant Blue (containing 40% methanol and 7% acetic acid). Areas of degradation were measured using an image analyzer (as clear bands against a darkly stained background) (Fuji-Film Corporation). Band densities were determined using multi-gauge image analysis software (Multi Gauge v.3.0; Fuji-Film Corporation).

### RT-qPCR

RNA was isolated from cells using TRIzol reagent (RNAiso Plus; Takara Bio, Inc., Shiga, Japan) and extracted using a FastPure RNA Kit (Takara Bio, Inc.). cDNA was synthesized using a PrimeScript RT reagent Kit (Takara Bio, Inc.) with heating at 37 °C for 15 min and then 85 °C for 5 s. mRNA levels were analyzed by qPCR using Power SYBR Green PCR Master Mix and the ABI PRISM 7900 sequence detection system (Applied Biosystems; Thermo Fisher Scientific.). The PCR amplification primers used were as follows: MMP-9 (NM 004994) CCTGGAGACCTGAGAACCAATCT (sense) and CCACCCGAGTGTAACCATAGC (antisense); SIRT6 (NM 001193285) CTTGGCACATTCTTCCACAA (sense) and GCTTCCTGGTCAGCCAGA (antisense); GAPDH (NM 002046) ATGGAAATCCCATCACCATCTT (sense) and CGCCCCACTTGATTTTGG (antisense). PCR was conducted over 40 cycles at 50 °C for 2 min, 95 °C for 10 min, 95 °C for 15 s, and 60 °C for 1 min. The data were normalized to GAPDH to control for differences in target mRNA concentration. Quantitation was conducted using the comparative Ct method^18^.

### Luciferase assay

Cells were seeded into 24-well plates and transfected with AP-1 or NF-κB reporter plasmids (provided by Professor Kim Chul Ho, SungKyunKwan University, Suwon, Korea) using Lipofectamine 2000 reagent (Invitrogen) according to the manufacturer’s instructions. Transfected cells were treated with TPA or TNF-α for 3 h. Luciferase reporter assays were conducted using the dual luciferase assay kit (Promega Corporation) according to the manufacturer’s instructions, and fluorescence intensities were measured using a luminometer (Lumat LB 9507, EG & G Berthold, Gaithersburg, MD, USA).

### Invasion assay

Invasion assays were conducted using 24-well chambers (8 μm pore size) in which the upper side of the Transwell insert was coated at 37 °C for 30 min with Matrigel (BD Biosciences, Franklin Lakes, NJ, USA). Cells were placed in the upper chambers, and the lower chambers were filled with conditioned medium containing TPA or TNF-α. After incubation for 24 h, the cells in the upper chambers were cleared using cotton swabs. The invaded cells on the bottom of the filter were fixed with 3.7–4.0% formalin for 10 min at room temperature and stained with crystal violet for 40 min at room temperature. Invading cells were counted in five randomly selected fields under a light microscope at 40 × magnification.

### Migration assay

Migration assays were also conducted using 24-well chambers (8 μm pore size). Migration assays were performed in chambers without Matrigel. Cells were delivered to the upper chambers, and the lower chambers were filled with conditioned medium containing TPA or TNF-α. After incubation for 24 h, cells in the upper chambers were cleared using cotton swabs. The migrated cells on the bottom of the filter were fixed with 3.7%–4.0% formalin for 10 min at room temperature and stained with crystal violet for 40 min at room temperature. Moving cells were counted in five random fields under a light microscope at 40 × magnification.

### Statistical analysis

Data from three independent experiments are presented as means ± standard error of the mean. Statistical analyses were conducted by Student’s t-test using Microsoft 2013 Excel (Redmond, USA). Statistical significance was accepted for *p* values < 0.05.

### Ethics approval

The biospecimens and data used in this study were provided by the Biobank of Jeonbuk National University Hospital, a member of the Korea Biobank Network, which is supported by the Ministry of Health, Welfare, and Family Affairs. All samples derived from the Korea Biobank Network were obtained with informed consent under institutional review board-approved protocols. This study was conducted after obtaining the approval of the Institutional Review Board of Jeonbuk National University Hospital (No. CUH 2020-12-010-002).

## Results

### MMP-9 and SIRT6 expression in human breast cancer tissue was higher than normal tissue

**Figure 1:**
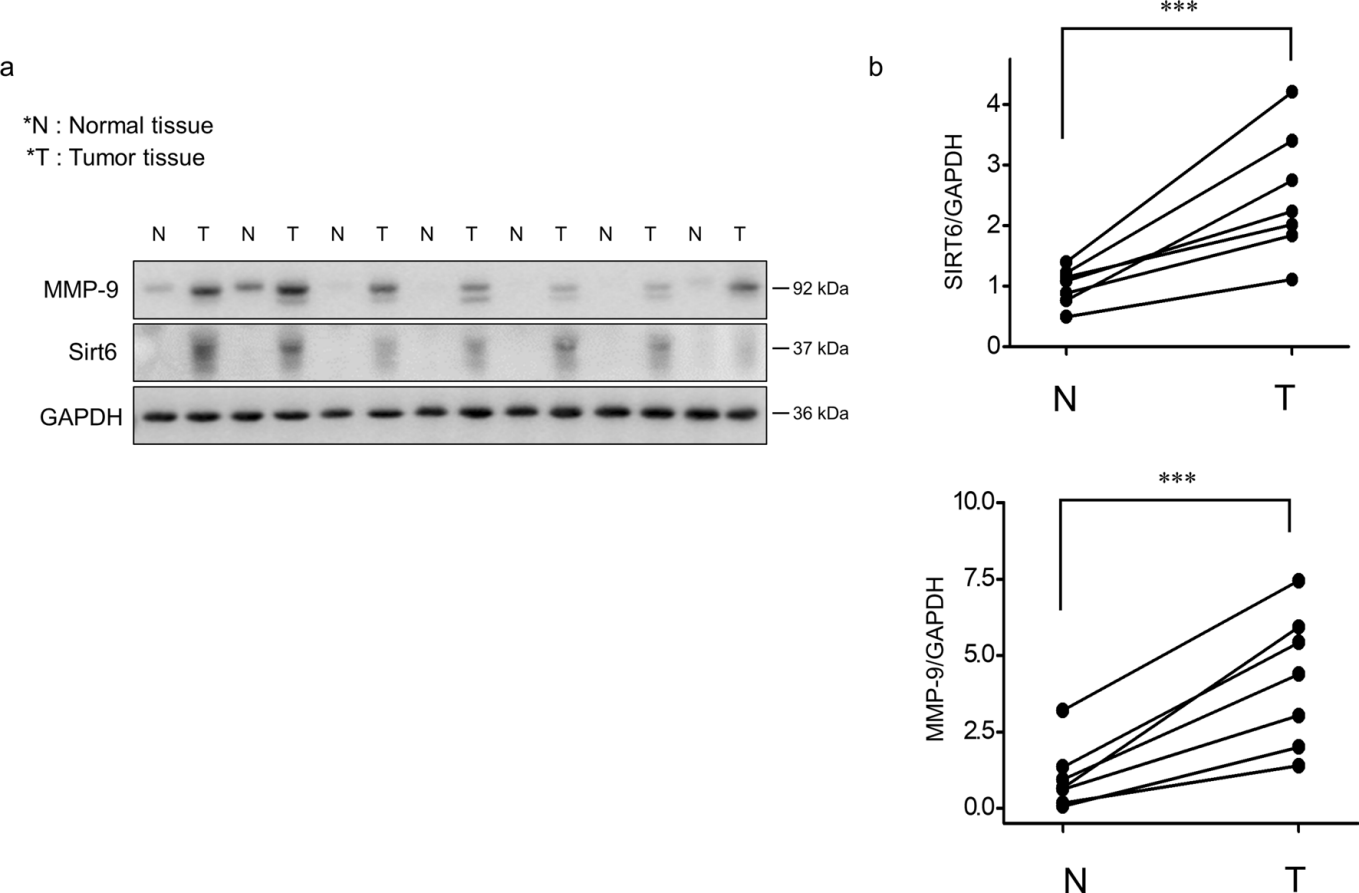
MMP-9 and SIRT6 expression in human breast cancer tissue and normal tissue. (**a**) Western blot analysis of MMP-9 and SIRT6 expression in normal (N) and breast cancer (T) tissues. GAPDH was used as a loading control. (**b**) Relative abundance of MMP-9 and SIRT6 levels in (**a**). (***p < 0.005). *N* normal breast tissue, *T* breast cancer tissue.

To assess MMP-9 and SIRT6 expression in clinical patients, protein levels in breast cancer tissues were analyzed by western blotting. The results showed that SIRT6 and MMP-9 expression in all malignant tissues was significantly higher than that in paired normal breast tissues (Student's *t*-test, *p* < 0.005; Fig. 1a,b).

### TPA and TNF-α upregulated MMP-9 expression

**Figure 2:**
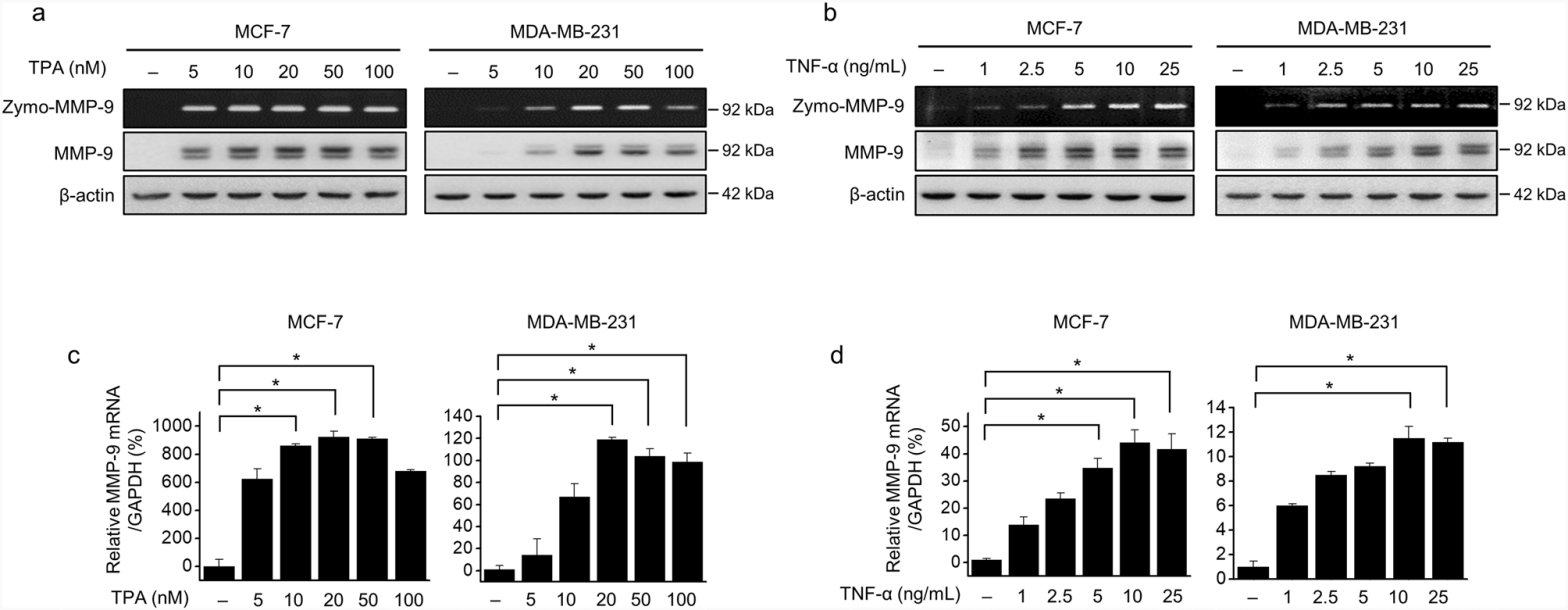
Upregulation of MMP-9 expression by TPA or TNF-α treatment. MCF-7 and MDA-MB-231 cells were treated with TPA or TNF-α for 24 h. (**a**, **b**) The amount of MMP-9 secreted into the medium was analyzed by gelatin zymography (zymo-MMP-9). MMP-9 levels were analyzed by western blotting using β-actin as an internal control. (**c**, **d**) *MMP-9* mRNA levels were examined by RT-qPCR, using *GAPDH* as an internal control. The results are presented as means ± standard error of three independent experiments. *p < 0.05, vs. untreated controls. *RT-qPCR* reverse transcription quantitative polymerase chain reaction.

MCF-7 and MDA-MB-231 cells were treated for 24 h with various concentrations of TPA (0–100 nmol/L) or TNF-α (0–25 ng/mL). Gelatin zymography (Zymo-MMP-9) showed that TPA- and TNF-α-induced MMP-9 secretion in media in a dose-dependent manner, and western blotting showed that TPA and TNF-α dose-dependently induced MMP-9 protein expression (Fig. 2a,b). Moreover, RT-qPCR showed that TPA and TNF-α dose-dependently induced MMP-9 mRNA levels (Fig. 2c,d). Therefore, treatment of MCF-7 and MDA-MB-231 cells with TPA or TNF-α significantly upregulated MMP-9 protein secretion as well as MMP-9 mRNA levels.

### SIRT6 upregulated TPA- or TNF-α-induced MMP-9 expression

**Figure 3:**
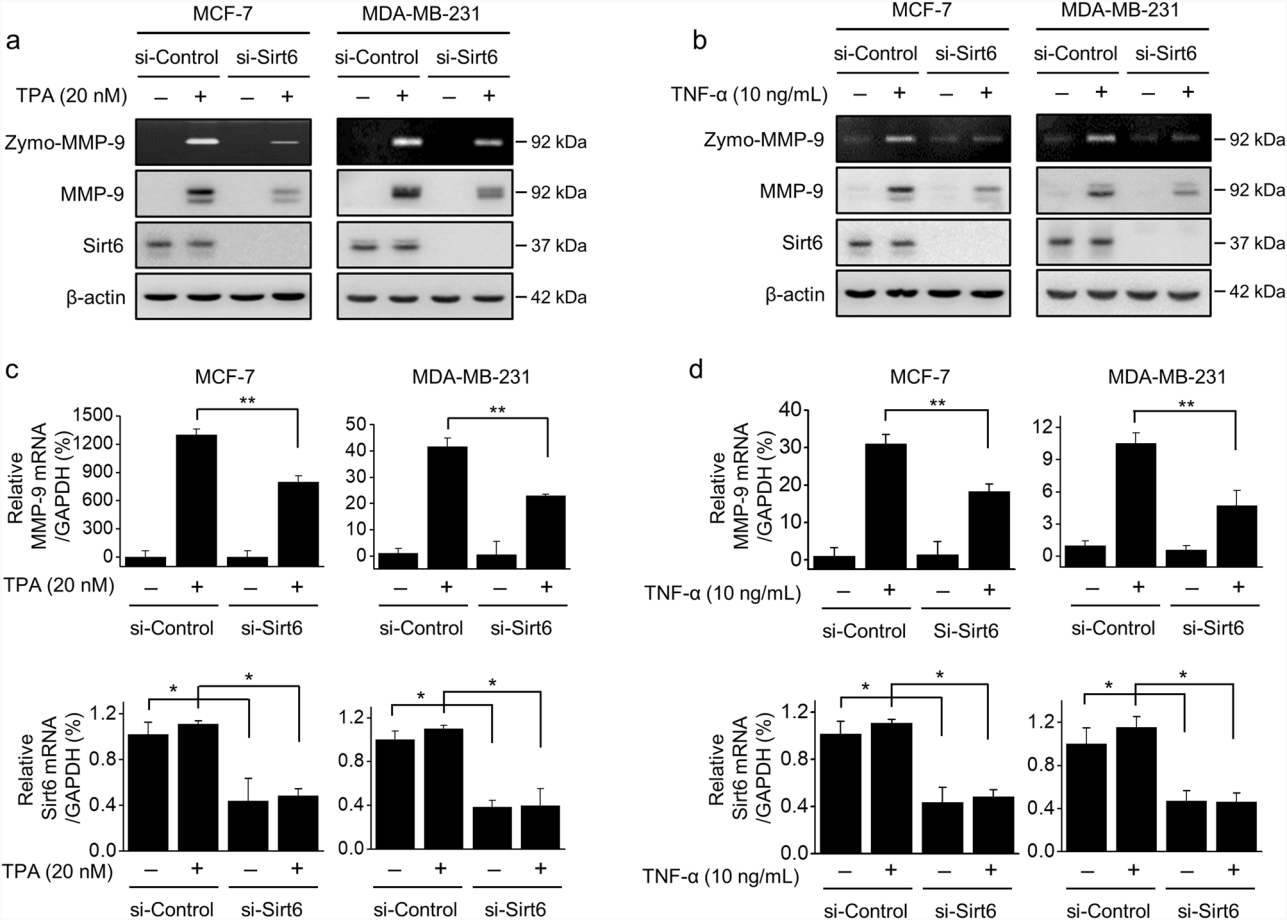
Effect of SIRT6 on MMP-9 expression. MCF-7 and MDA-MB-231 cells were transfected with control siRNA or *SIRT6* siRNA and treated with TPA or TNF-α for 24 h. (**a**, **b**) MMP-9 secretion into the medium was analyzed by gelatin zymography (zymo-MMP-9). MMP-9 and SIRT6 levels were examined by western blotting using β-actin as an internal control. (**c**, **d**) *MMP-9* and *SIRT6* mRNA levels were examined by RT-qPCR, using *GAPDH* as an internal control. The results are presented as means ± standard error of three independent experiments. *p < 0.05, vs. TPA- or TNF-α-treated control siRNA; **p < 0.01 vs. TPA- or TNF-α-treated control siRNA.

To investigate the effect of SIRT6 on TPA- or TNF-α-induced MMP-9 expression, MCF-7 and MDA-MB-231 cells were subjected to siRNA-mediated silencing of SIRT6. The TPA- or TNF-α-induced elevated MMP-9 secretion and protein levels in MCF-7 and MDF-MB-231 cells were significantly reduced by SIRT6 knockdown (Fig. 3a,b). In addition, SIRT6 knockdown reduced TPA- or TNF-α-induced MMP-9 mRNA expression in both cell lines (Fig. 3c,d).

**Figure 4:**
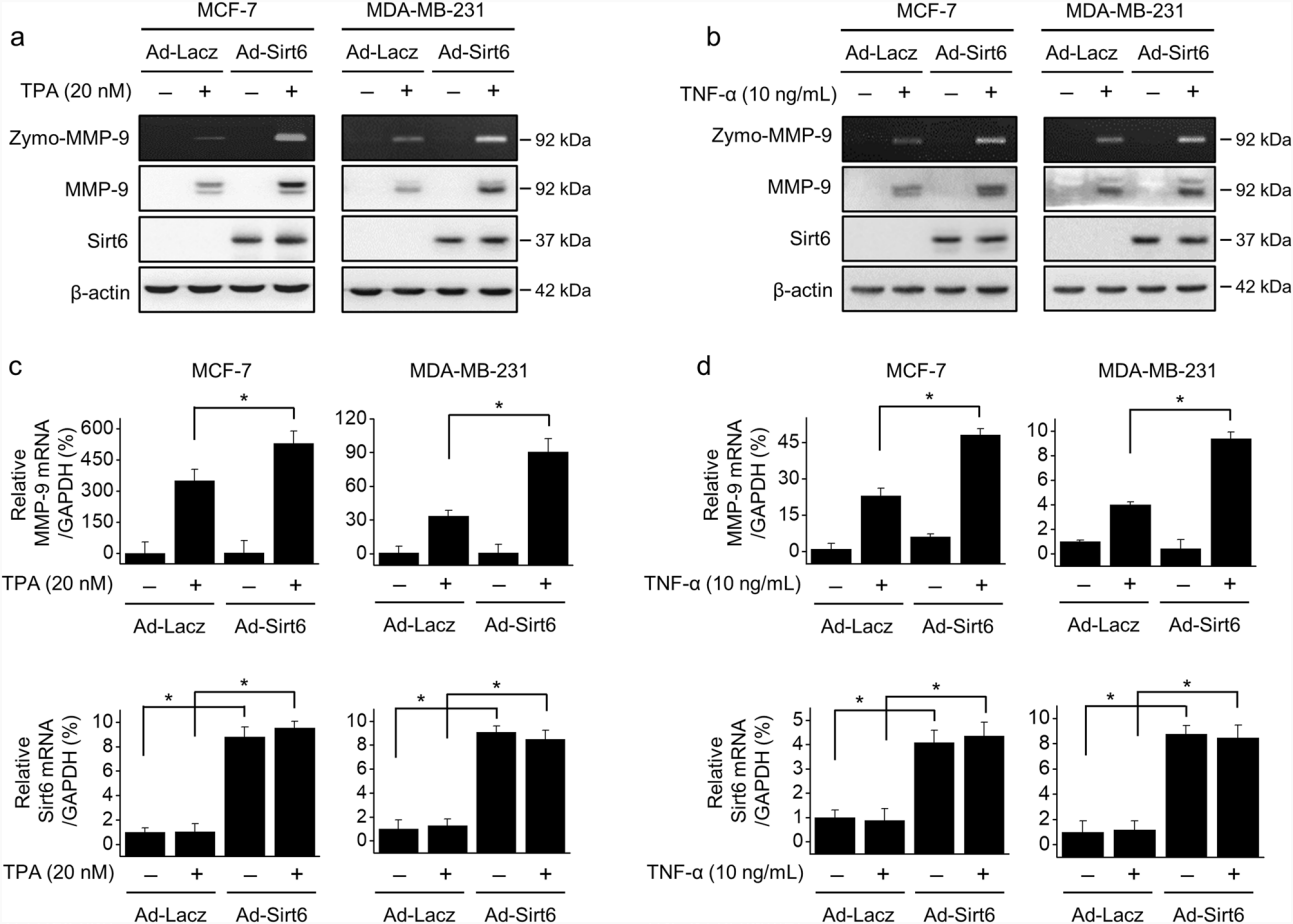
Effect of SIRT6 overexpression on MMP-9 expression. MCF-7 and MDA-MB-231 cells were infected with adeno-*SIRT6* virus or adeno-*LacZ* virus and then treated with TPA or TNF-α for 24 h. (**a**, **b**) MMP-9 secretion in the medium was examined by gelatin zymography (zymo-MMP-9). MMP-9 and SIRT6 protein levels were examined by western blotting using β-actin as an internal control. (**c**, **d**) *MMP-9* and *SIRT6* mRNA levels were analyzed by RT-qPCR, using *GAPDH* as an internal control. The results are presented as means ± standard error of three independent experiments. *p < 0.05 vs. TPA- or TNF-α-treated adeno-*Lac*Z virus.

To further investigate the modulating effect of SIRT6 on MMP-9 expression, MCF-7 and MDA-MB-231 cells were infected with the adeno-SIRT6 virus or adeno-LacZ virus. SIRT6 overexpression treatment in TPA- or TNF-α-induced MMP-9 secretion and protein expression additively upregulated MMP-9 expression (Fig. 4a,b) and MMP-9 mRNA levels (Fig. 4c,d) in both cell lines. These results confirmed the involvement of SIRT6 in MMP-9 expression.

### SIRT6 knockdown reduced TPA- or TNF-α-induced MMP-9 expression by inhibiting MAPK phosphorylation

PKC is known to be important in the signal transduction of MMP-9 activation^10^. To confirm the involvement of PKC activation, we performed membrane fractionation in TPA- or TNF-α-treated MCF-7 and MDA-MB-231 cells. Cells were separated into cytosolic and membrane fractions to investigate their activity. TPA or TNF-α caused the translocation of PKCα, PKCβ, and PKCδ from the cytosol to the membrane in both cell lines. However, silencing of SIRT6 did not alter localization of PKC isoforms (Supplementary Fig. 1).

**Figure 5:**
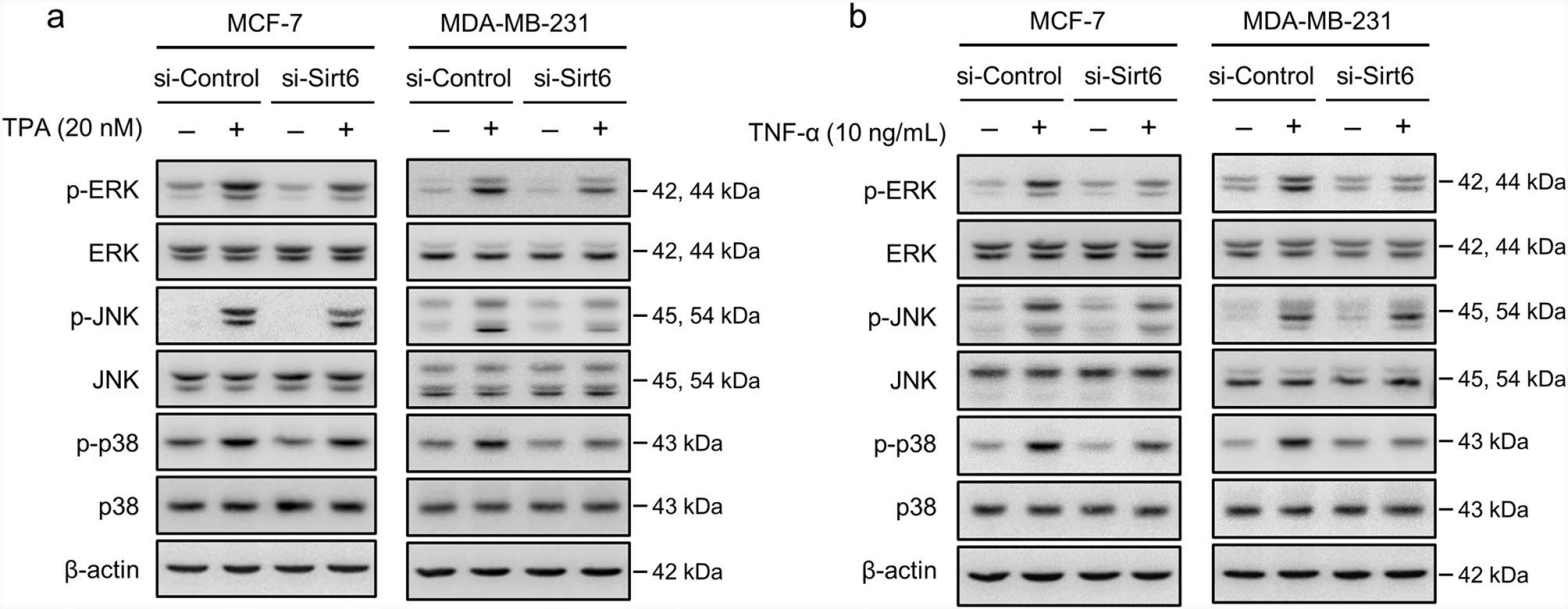
Effect of SIRT6 on MAPK. (**a**, **b**) MCF-7 and MDA-MB-231 cells transfected with control siRNA or *SIRT6* siRNA were treated with TPA or TNF-α for 30 min. Phosphorylated ERK, JNK, and p38 levels and total ERK, JNK, and p38 levels were examined by western blotting. β-actin was used as an internal control.

To investigate the molecular mechanisms responsible for TPA- or TNF-α-induced activation of MMP-9, MCF-7 and MDA-MB-231 cells were pretreated with pharmacological inhibitors of MAPK signaling pathways. The MAPK inhibitors used were PD98059 (ERK inhibitor), SP600125 (JNK inhibitor), and SB203580 (p38 inhibitor). TPA- or TNF-α-induced MMP-9 secretion and protein expression were reduced by each 20 μM concentration of three MAPK inhibitors in both cell lines (Supplementary Fig. 2a,b). We investigated the effects of SIRT6 on the MAPK signaling pathway in MCF-7 and MDA-MB-231 cells. Additionally, TPA significantly increased the phosphorylation of ERK, JNK, and p38 in both cell lines, and SIRT6 knockdown decreased the phosphorylation of ERK and JNK in MCF-7 cells, and inhibited the phosphorylation of ERK, JNK, and p38 in MDA-MB-231 cells; however, total protein levels remained unaltered (Fig. 5a). TNF-α increased the phosphorylation of ERK, JNK, and p38 in both cell lines, and SIRT6 knockdown decreased the phosphorylation of ERK, JNK, and p38 in MCF-7 cells, and reduced the phosphorylation of ERK and p38 in MDA-MB-231 cells; however, total protein levels remained unaltered (Fig. 5b). These results showed that silencing of SIRT6 reduced phosphorylation of MAPK and reduction of MAPK activity correlated with reduced MMP-9 expression.

### SIRT6 knockdown suppressed TPA- or TNF-α-induced MMP-9 expression by reducing the activation of NF-κB and AP-1

**Figure 6:**
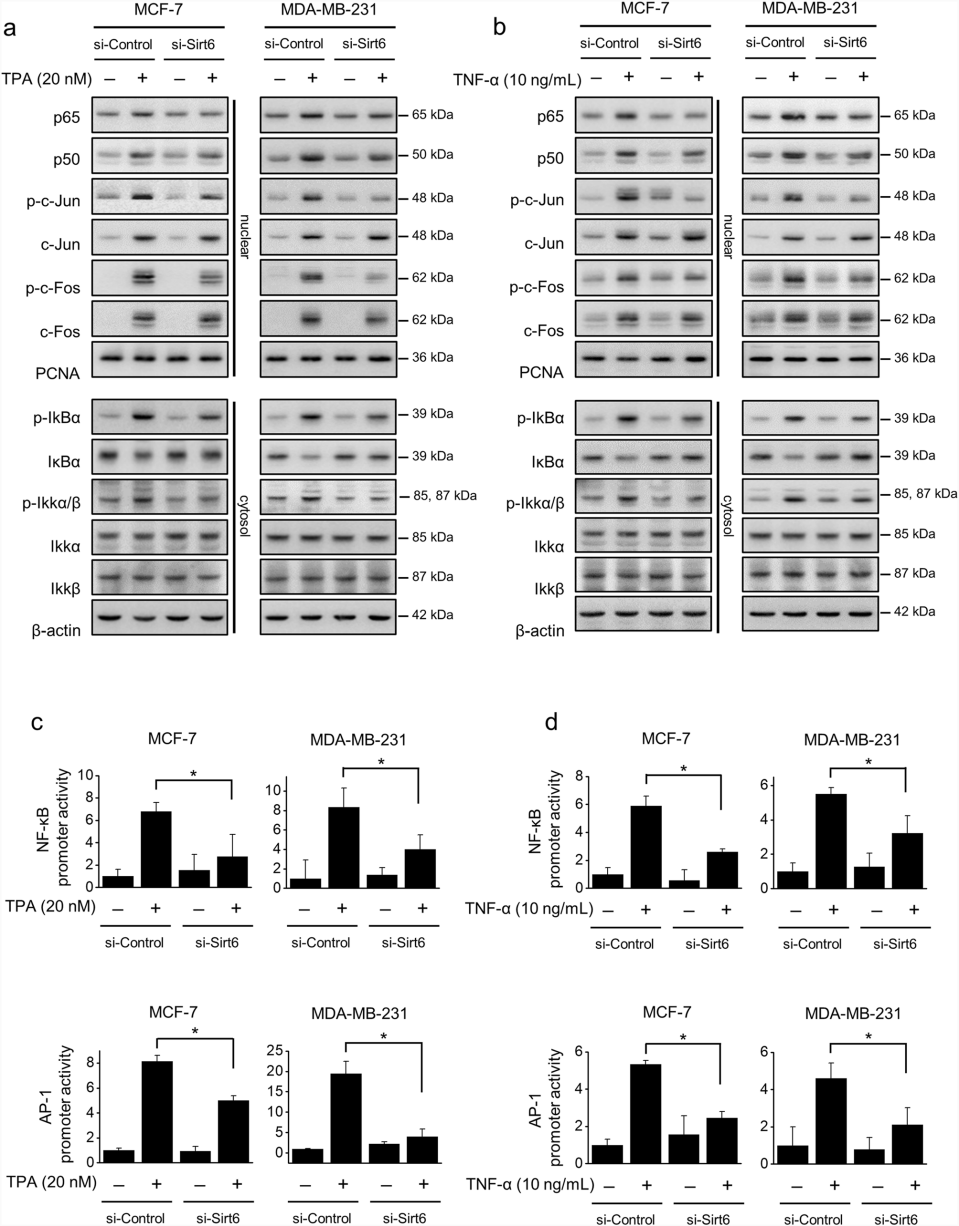
Effects of SIRT6 on NF-κB and AP-1 activation. (**a**, **b**) MCF-7 and MDA-MB-231 cells were transfected with control siRNA or *SIRT6* siRNA and then treated with TPA or TNF-α for 3 h. Cytosolic and nuclear extracts were prepared and nuclear protein extracts were analyzed by western blotting using antibodies against p65, p50, p–c-Fos, p–c-Jun, c-Fos, and c-Jun, and cytosolic protein extracts were examined by western blotting using antibodies against IκBα, p-IκBα, IKKα, IKKβ, and p-IKKα/β. PCNA was used as a nuclear loading control, and β-actin was used as an internal control for cytoplasmic protein detection. (**c**, **d**) NF-κB-luc and AP-1-luc reporters were co-transfected with luciferase thymidine kinase (*Renilla*) reporter into both cell lines. Thereafter, cells were transfected with control siRNA or *SIRT6* siRNA and treated with TPA or TNF-α for 4 h. NF-κB and AP-1 promoter activities were examined using a dual-luciferase reporter assay. The results are presented as means ± standard error of three independent experiments. *p < 0.05, vs. TPA- or TNF-α-treated control siRNA. *Renilla* Renilla luciferase thymidine kinase reporter.

To further understand the mechanisms responsible for MMP-9 transcriptional regulation, MCF-7 and MDA-MB-231 cells were pretreated with inhibitors of NF-κB (Bay 11-7092) or AP-1 (SR 11302) to examine the effects of MMP-9 on the activation of NF-κB and AP-1. Inhibition of NF-κB or AP-1 blocked TPA- or TNF-α-induced increases in MMP-9 secretion and protein levels (Supplementary Fig. 2c,d). In addition, the effects of SIRT6 on the activation of NF-κB and AP-1 were investigated in MCF-7 and MDA-MB-231 cells. Nuclear translocation of p65 and p50 (subunits of NF-κB) and phosphorylation of c-Jun and c-Fos (subunits of AP-1), as well as cytoplasmic phosphorylation of IKKα/β and degradation of IκBα (a subunit of NF-κB), were increased by TPA treatment. SIRT6 knockdown reduced the nuclear translocation of p65, p50, p-c-Jun, and p-c-Fos and reduced the cytoplasmic levels of p-IKKα/β and degradation of IκBα. Total c-Fos and c-Jun levels in the nucleus and total IKKα and IKKβ levels in the cytosol did not exhibit any changes (Fig. 6a). SIRT6 knockdown suppressed the TNF-α-induced nuclear translocation of p65, p50, p-c-Jun, and p-c-Fos, and inhibited the phosphorylation of cytoplasmic IKKα/β and degradation of IκBα. Total c-Fos and c-Jun in the nucleus and total IKKα and IKKβ in the cytosol did not exhibit any changes (Fig. 6b). The MMP-9 promoter consists of AP-1- and NF-κB binding sites; these transcription factors participate in the activation of the MMP-9 gene by TPA or TNF-α treatment. Luciferase assays for evaluation of the transactivation activities of NF-κB and AP-1 after TPA or TNF-α treatment showed that these interactions were significantly reduced in SIRT6-knockdown cells (Fig. 6c,d). These results suggested that SIRT6 knockdown suppressed MMP-9 expression by reducing the activation of NF-κB and AP-1.

### SIRT6 knockdown suppressed TPA- or TNF-α-induced Matrigel invasion and chamber migration

**Figure 7:**
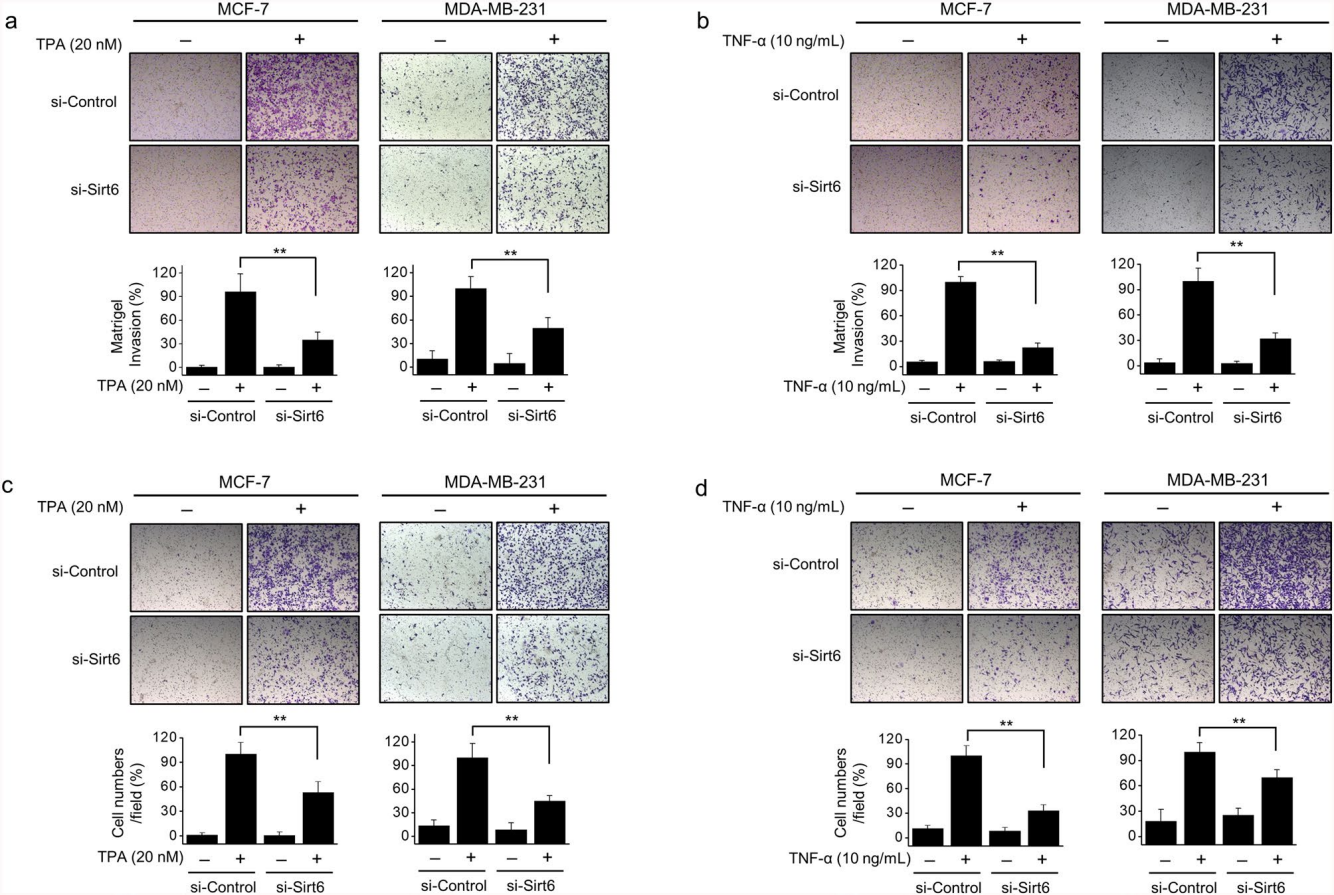
*SIRT6* knockdown inhibited TPA- and TNF-α-induced Matrigel cell invasion and migration. (**a**, **b**) MCF-7 and MDA-MB-231 cells were transfected with control siRNA or *SIRT6* siRNA and subjected to Matrigel invasion assays. The chambers were coated with Matrigel. Cells were seeded into the upper chambers, treated with TPA or TNF-α for 24 h, and examined under a light microscope at 40 × magnification. (**c**, **d**) MCF-7 and MDA-MB-231 cells were transfected with control siRNA or *SIRT6* siRNA and subjected to migration assays. The migration chamber was then implemented without Matrigel. Cells were seeded into the upper chambers, and TPA or TNF-α was added to the lower chambers. After treatment for 24 h, the cells were photographed under a light microscope at 40 × magnification. The results are shown as the mean number of migrated cells, and were obtained by counting migrated cells in five randomly selected fields. The results are presented as means ± standard error of three independent experiments. **p < 0.01 vs. TPA- or TNF-α-treated control siRNA.

We investigated the effects of SIRT6 on the capability of MCF-7 and MDA-MB-231 cells to degrade matrigel using Matrigel Transwell assays. The results showed that TPA and TNF-α significantly increased cell invasion, and SIRT6 knockdown significantly reduced the TPA- or TNF-α-induced increase in cell invasion in both cell lines (Fig. 7a,b). A migration assay was performed in a chamber without Matrigel. While determining the involvement of SIRT6 in breast cancer cell migration, SIRT6 knockdown was found to reduce TPA- or TNF-α-induced cell migration in both cell lines (Fig. 7c,d). These results showed that SIRT6 suppressed the malignant invasion and migration potential of MCF-7 and MDA-MB-231 cells.

## Discussion

Breast cancer is the most common cancer in the world and the leading cause of cancer death in female. Regulation of metastasis in breast cancer has been a major goal for successful treatment because most breast cancer-related deaths are due to advanced disease and progressive metastasis^19^. Cancer cell metastasis is a multi-step process that requires tumor cell invasion, migration to the circulation, extravasation, and growth in the metastatic region^20^. Invasion and migration are considered to be the most critical factors in primary tumor metastasis^3^. Here, we obtained the novel findings that SIRT6 is involved in the modulation of breast cancer cell invasion and migration by regulating MMP-9 expression in breast cancer cells, suggesting that SIRT6 is a novel target molecule for the prevention of breast cancer.

Sirtuins play key roles in various biological processes, including tumor progression. Seven sirtuins (SIRT1 to SIRT7) have been identified^21^, and SIRT6 has been reported to participate in tumor suppression and promotion. Tumor promotion by SIRT6 has been reported in several types of malignancies^22^; however, further studies are required to determine the role of SIRT6 in cancer invasiveness. In breast cancer cells, SIRT6 has been related to the upregulation of MMP-9^20^, and SIRT6 knockdown has been shown to reduce MMP-9 expression^21^. In our study, SIRT6 additively upregulated TPA- and TNF-α-induced MMP-9 expression and silencing of SIRT6 reduced TPA- and TNF-α-induced MMP-9 expression (Figs. 3 and 4). These data showed that SIRT6 involved in MMP-9 expression. MMP-9 is a major member of the zinc metalloproteinase family because it stimulates cancer metastasis by degrading ECM^23^ and collagens, facilitating cancer cell invasion and metastasis^24^. Therefore, MMP-9 is a potentially key molecule in cancer invasion^25^ and is considered a target for drug development^26^. The results obtained in the present study revealed that SIRT6 is involved in the modulation of breast cancer cell invasion and migration by regulating MMP-9 expression in MCF-7 and MDA-MB-231 cells.

The synthesis and secretion of MMP-9 are strictly regulated by various biological factors^27^. Several studies have reported that TPA activates the synthesis and secretion of MMP-9 in breast cancer cells^28^. TPA induces inflammatory responses and acts as a tumor promoter that stimulates PKC isozymes by direct binding. TPA dramatically induces the invasion of human breast cancer cells by upregulating MMP-9 expression via transcription factors and MAPK pathways^29^. In addition, TNF-α is known to increase tumor cell migration and invasion, and is involved in all steps of tumorigenesis^30^. TNF-α-induced tumor initiation and tumor promotion are mediated by MMP-9-induced activation of NF-κB and AP-1-dependent signals in tumor cells^31^. Our study showed that TPA and TNF-α significantly upregulated MMP-9 expression in MCF-7 and MDA-MB-231 cells (Fig. 2). SIRT6 regulate the fatty acylation level of TNF-α and then the synthesis of TNF-α^32^. This study also suggested that SIRT6 upregulated TPA and TNF-α expression in MCF-7 and MDA-MB-231 cells (Figs. 3 and 4).

PKC activity is important for cancer cell migration and is correlated with MMP-9 expression in breast cancer cells. Our data showed that TPA or TNF-α mediated PKC activity, but we identified no associations involving PKC isoforms and SIRT6 in breast cancer cells (Supplementary Fig. 1). Thus, we focused on other mechanisms related to cancer progression and metastasis.

MMP-9 promoters have various transcription factor-binding sites, and AP-1 and NF-κB are key transcription factors^8^. Nuclear factors binding to the AP-1 and NF-κB promoters are induced and synergistically contributed by TPA and TNF-α in tumor cell invasiveness^33^. TNF-α activates the receptor tyrosine kinase pathway, leads to activation of NF-κB. And the AP-1 transcription factor is known to be key for MMP-9 expression. In addition, the downregulated p65 blocked TNF-α-induced MMP-9 expression and increase of IκBα blocked NF-κB activity and MMP-9 expression. This demonstrates that NF-κB activity is essential for upregulating MMP-9 expression^34^. Inhibition of MMP-9 and cell invasion involves inhibition of the MAPK pathway^35^. SIRT6 is known to regulate the activation of MAPKs signaling-correlated genes by deacetylation. However, the functional role of SIRT6 deacetylase activity in the MAPK signaling pathway is yet to be fully understood. SIRT6 positively modulated the levels of phosphorylated ERK and regulated MMP9 abundance probably through the MAPK signaling pathway^36^. Our data also showed that silencing of SIRT6 modulated activation of MAPK expression (p-ERK, p-JNK, p-p38). These results indicated that SIRT6 involved MAPK signaling pathway. In order to better understand the signaling cascades underlying MMP-9 expression and SIRT6 modulation in MCF-7 and MDA-MB-231 cells, the effects of specific inhibitors of MAPKs, NF-κB, and AP-1 were assessed. MMP-9 expression were significantly inhibited by MAPKs, NF-κB, and AP-1 (Supplementary Fig. 2). In addition, the transactivation activities of NF-κB and AP-1 were reduced, and MAPK phosphorylation was suppressed by SIRT6 knockdown; therefore, MMP-9 expression was downregulated. These results showed that MMP-9 is mainly regulated by the MAPK, NF-κB, and AP-1 signaling pathways, although it should be noted that not all MAPKs are involved (Figs. 5 and 6). The anti-invasive effect of SIRT6 can be attributed to the suppression of MAPK signaling, NF-κB and AP-1 activation, and MMP-9 expression.

In conclusion, SIRT6 regulated the migration and invasion of breast cancer cells in vitro and played an essential role in TPA- or TNF-α-induced MMP-9 expression. Furthermore, SIRT6 knockdown suppressed TPA- or TNF-α-induced MMP-9 expression by inhibiting the MAPK, AP-1, and NF-κB signaling pathways in MCF-7 and MDA-MB-231 cells. This study suggests that SIRT6 is a novel target molecule for the prevention of breast cancer invasion and metastasis.

## Supplementary Information

Supplementary Legends.

Supplementary Information.

Supplementary Figure 1.

Supplementary Figure 1.

Supplementary Figure 2.

Supplementary Figure 2.

Supplementary Figure 2.

Supplementary Figure 2.

Supplementary Figure 2.

## Data Availability

The datasets used and analyzed in this study are available from the corresponding author upon reasonable request.
