# Supplementary material for: Inhibition of cell invasion and migration by targeting matrix metalloproteinase-9 expression via sirtuin 6 silencing in human breast cancer cells
Source: Sci Rep. 2022 Jul 15;12:12125. doi: 10.1038/s41598-022-16405-x (PMC9287314; doi:10.1038/s41598-022-16405-x)
Supplement: Supplementary file 1 — Supplementary Legends. [file 41598_2022_16405_MOESM1_ESM.docx]

**Supplement figure legends**

**Fig. 1 Effect of SIRT6 on PKC activity**

**a and b** MCF-7 and MDA-MB-231 cells transfected with control siRNA or SIRT6 siRNA were treated with TPA or TNF-α for 40 min. Cytosolic and membrane extracts were prepared, and cytosolic and membrane protein extracts were analyzed by western blotting using antibodies against PKCα, PKCβ, and PKCδ. β-actin was used as an internal control for cytoplasmic protein detection, and Na-K ATPase was used as a membrane protein loading control.

**Fig. 2 MMP-9 expression by TPA and TNF-α -induced through MAPK/NF-κB/AP-1**

**a and b** MCF-7 and MDA-MB-231 cells were pretreated with MAPK signaling inhibitors (i.e. PD98059 for ERK, SP600125 for JNK, or SB203580 for p38) for 1 h and then treated with TPA or TNF-α for 24 h. MMP-9 secretion into the medium was examined by gelatin zymography (zymo-MMP-9). MMP-9 levels were examined by western blotting using β-actin as an internal control. **c and d** MCF-7 and MDA-MB-231 cells were pretreated with inhibitors of NF-κB (Bay 11-7092) or AP-1 (SR 11302) and then treated with TPA for 24 h. MMP-9 levels in media were examined by gelatin zymography, and protein levels were analyzed by western blotting.
