# Supplementary figures and images for: Inhibition of cell invasion and migration by targeting matrix metalloproteinase-9 expression via sirtuin 6 silencing in human breast cancer cells
Source: Sci Rep. 2022 Jul 15;12:12125. doi: 10.1038/s41598-022-16405-x (PMC9287314; doi:10.1038/s41598-022-16405-x)

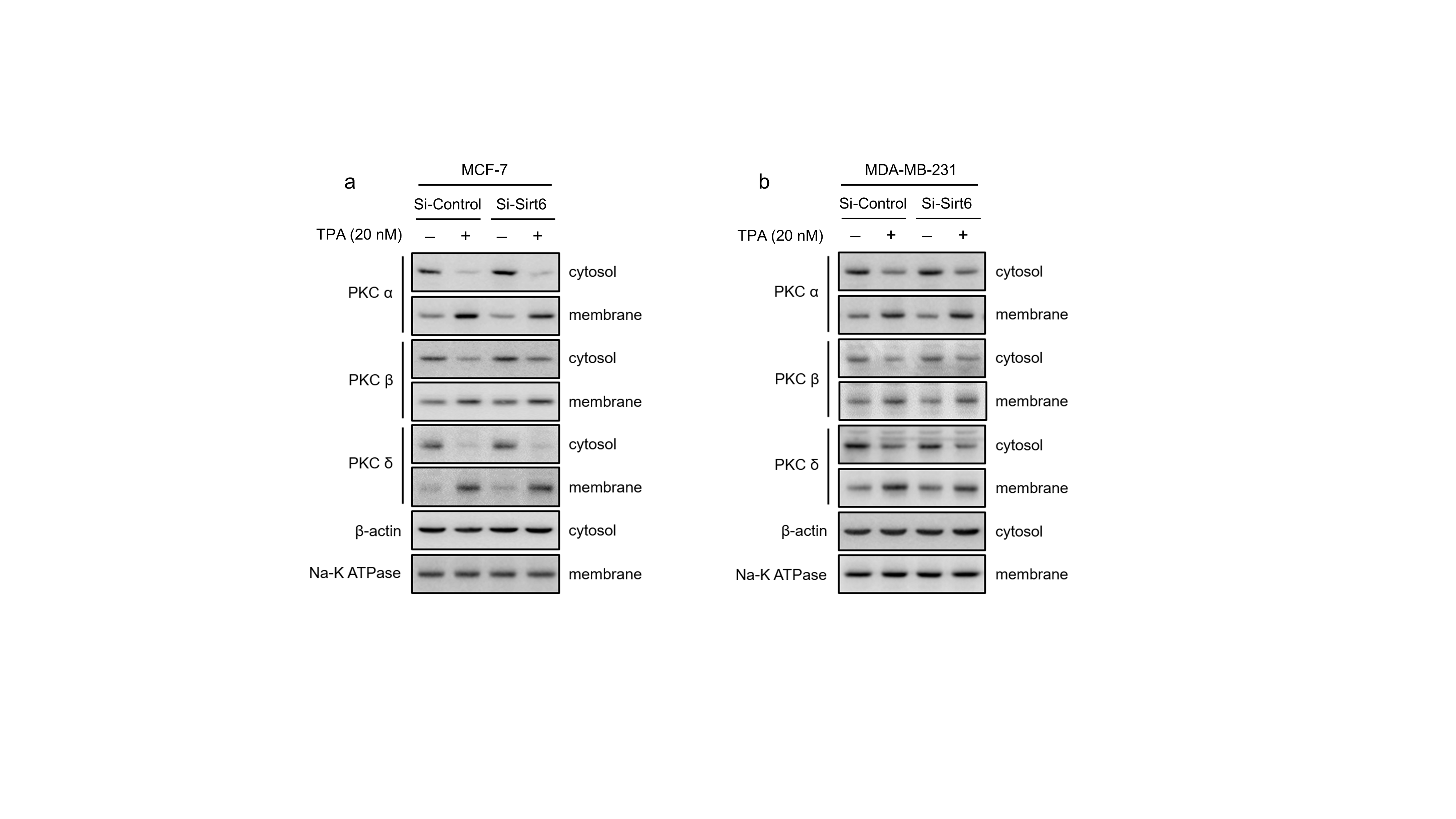

Supplement: Supplementary file 3 — Supplementary Figure 1. [file 41598_2022_16405_MOESM3_ESM.tif]

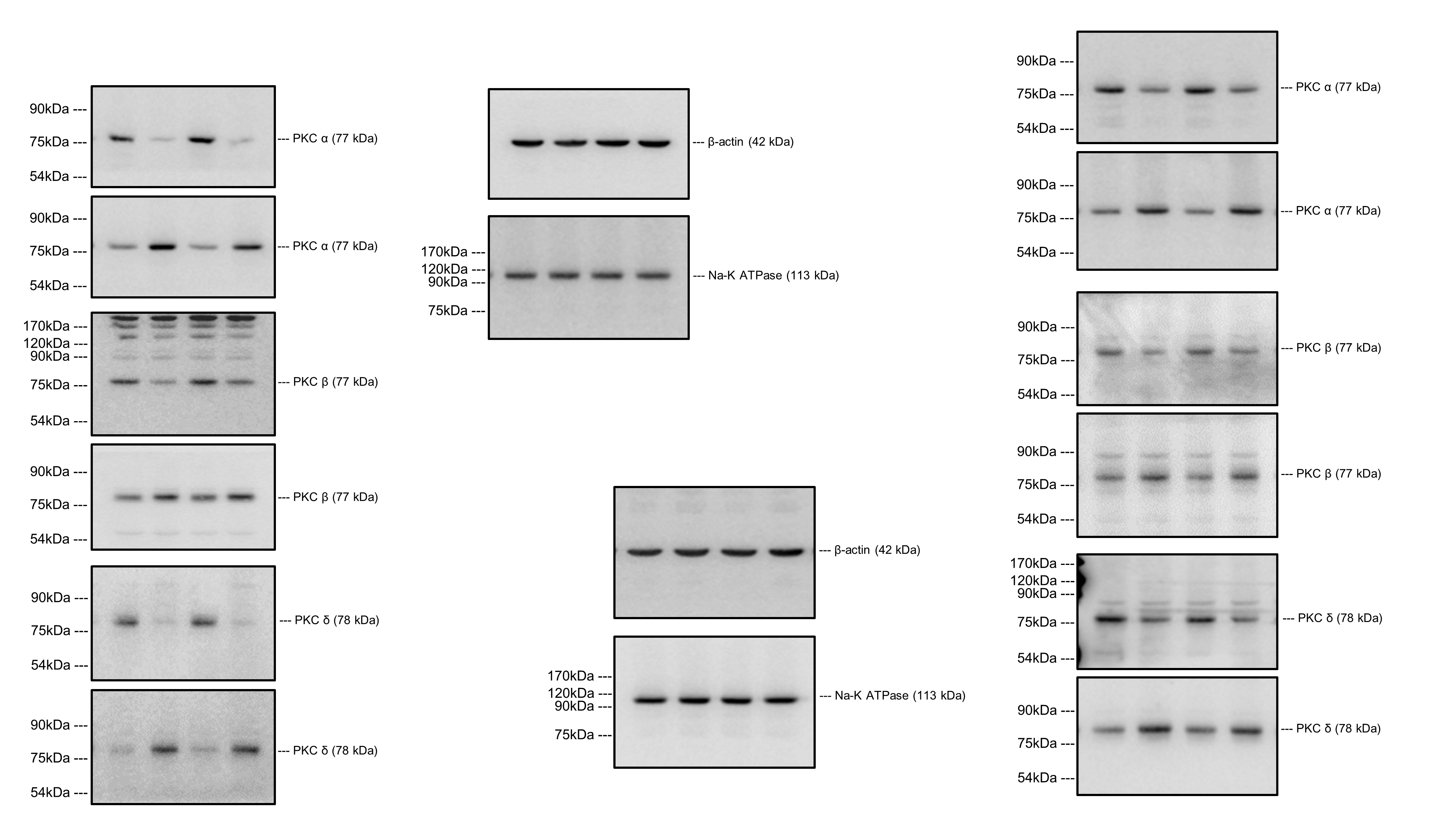

Supplement: Supplementary file 4 — Supplementary Figure 1. [file 41598_2022_16405_MOESM4_ESM.png]

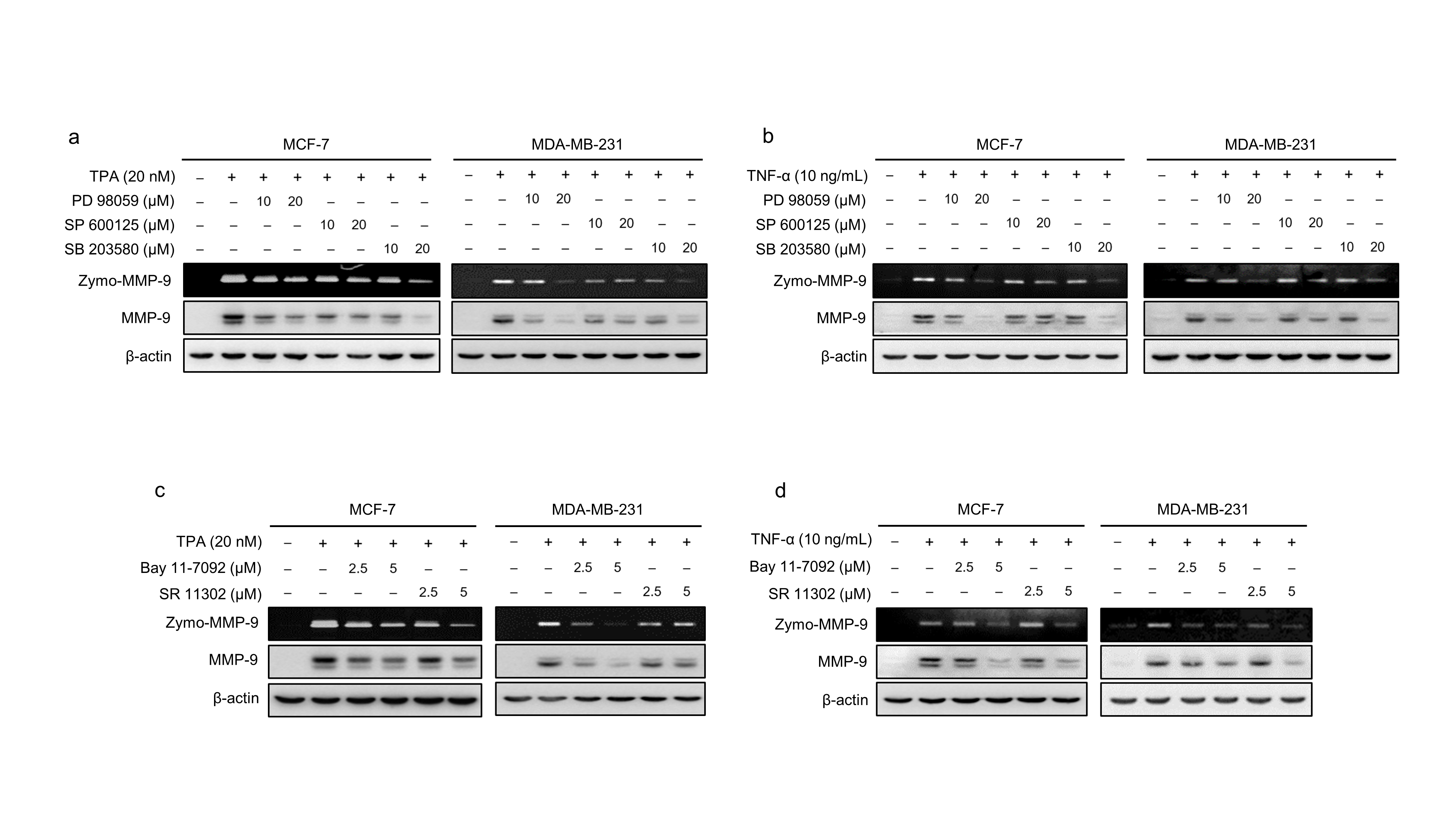

Supplement: Supplementary file 5 — Supplementary Figure 2. [file 41598_2022_16405_MOESM5_ESM.tif]

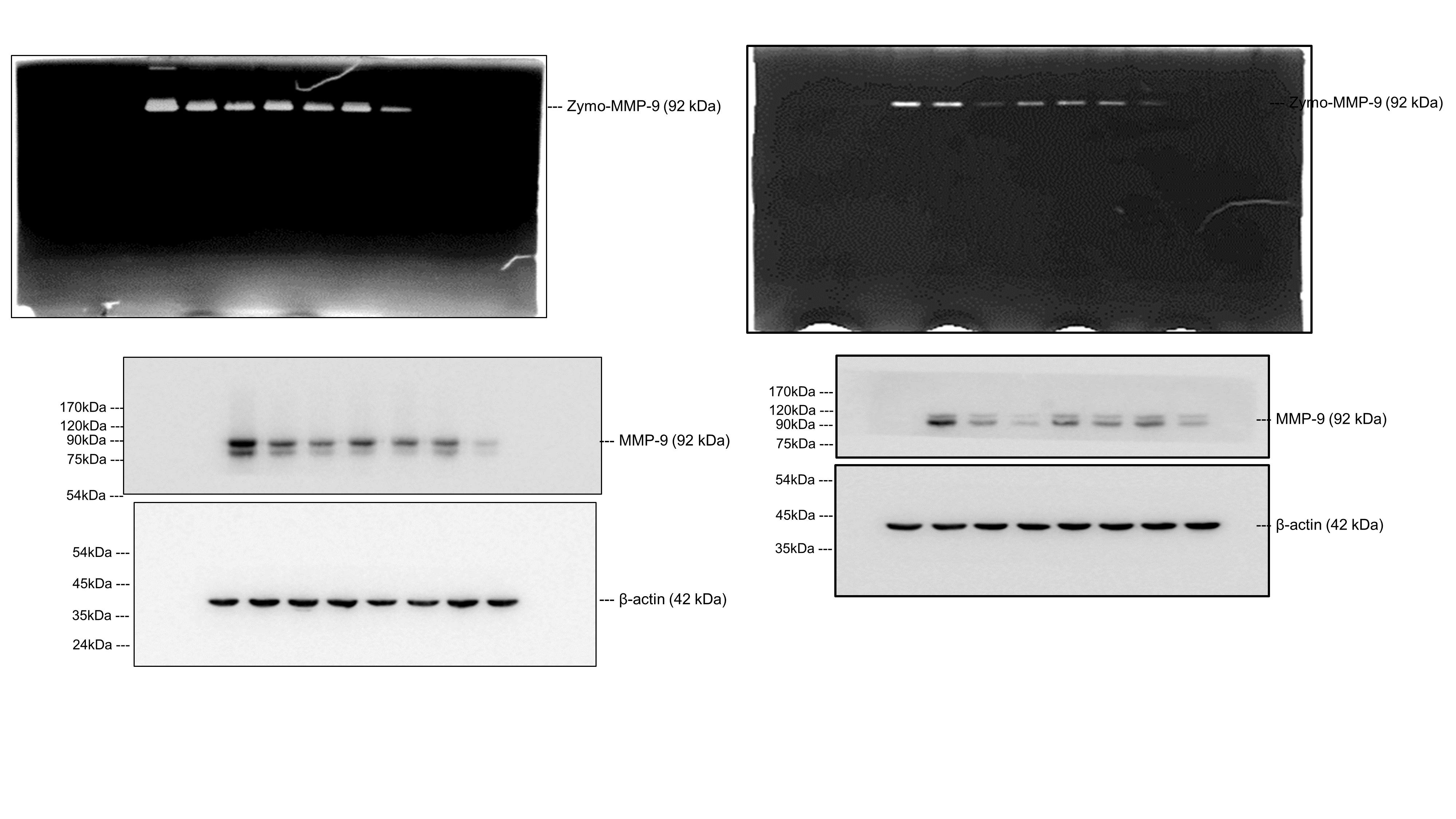

Supplement: Supplementary file 6 — Supplementary Figure 2. [file 41598_2022_16405_MOESM6_ESM.png]

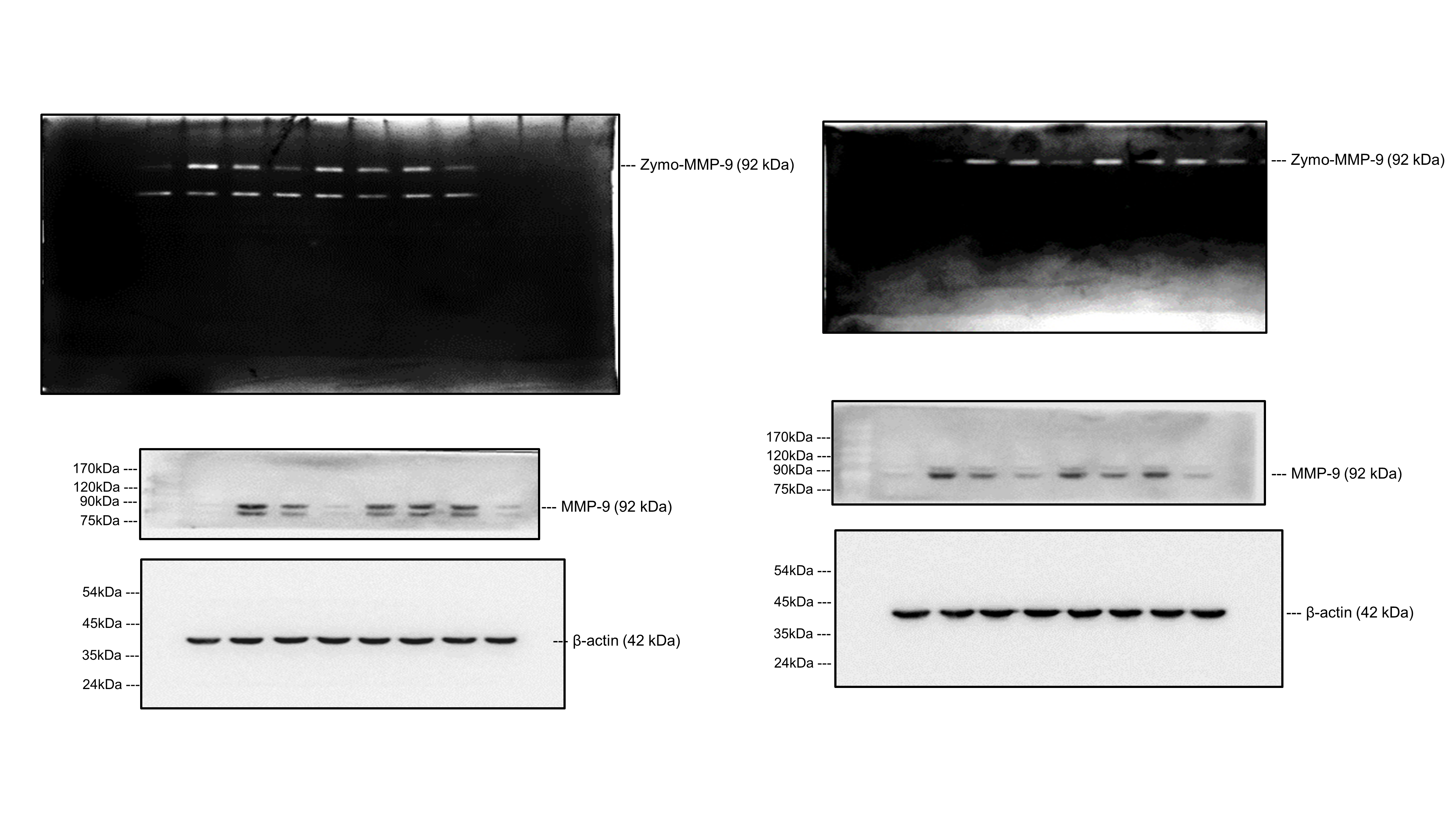

Supplement: Supplementary file 7 — Supplementary Figure 2. [file 41598_2022_16405_MOESM7_ESM.png]

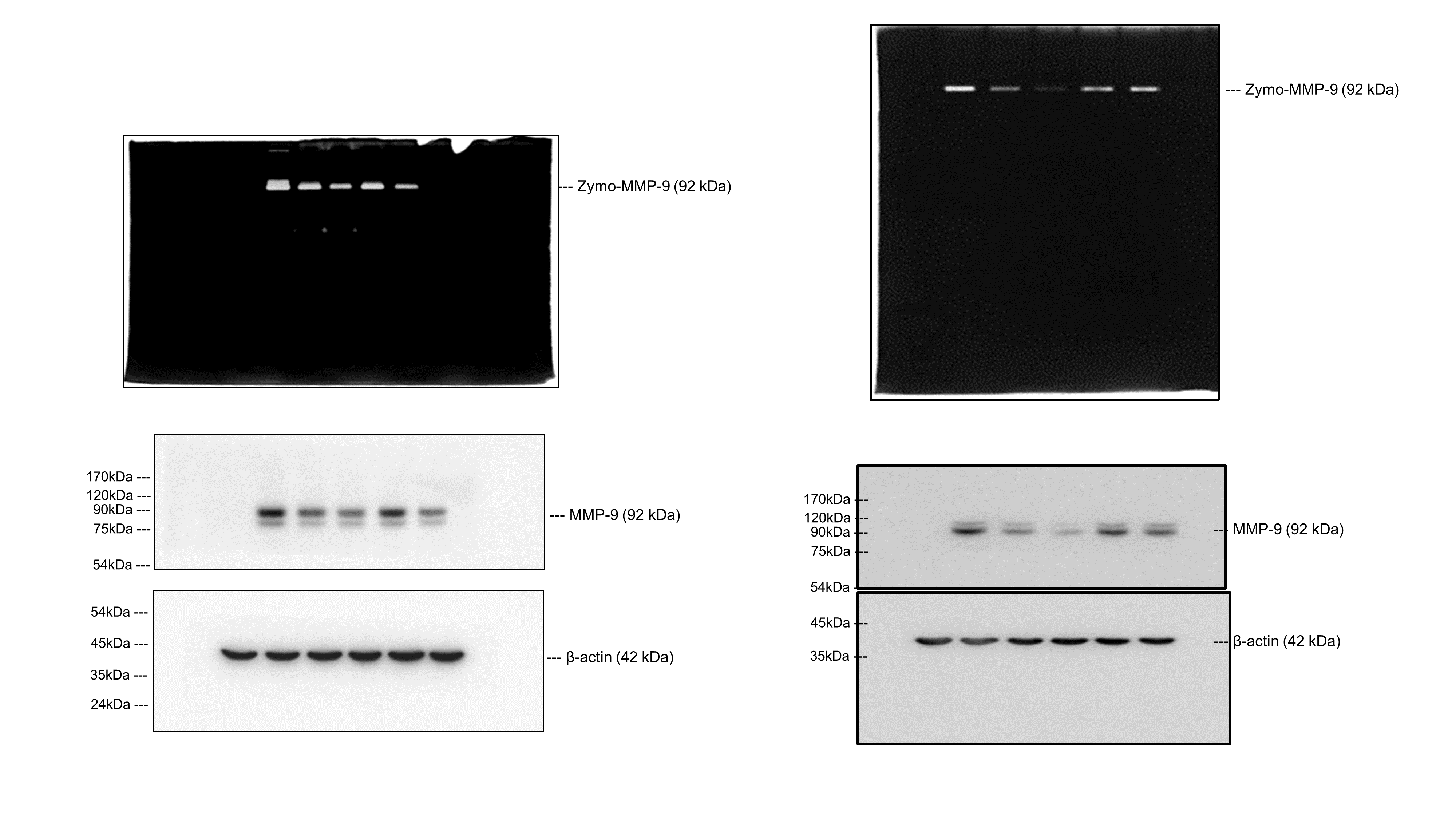

Supplement: Supplementary file 8 — Supplementary Figure 2. [file 41598_2022_16405_MOESM8_ESM.png]

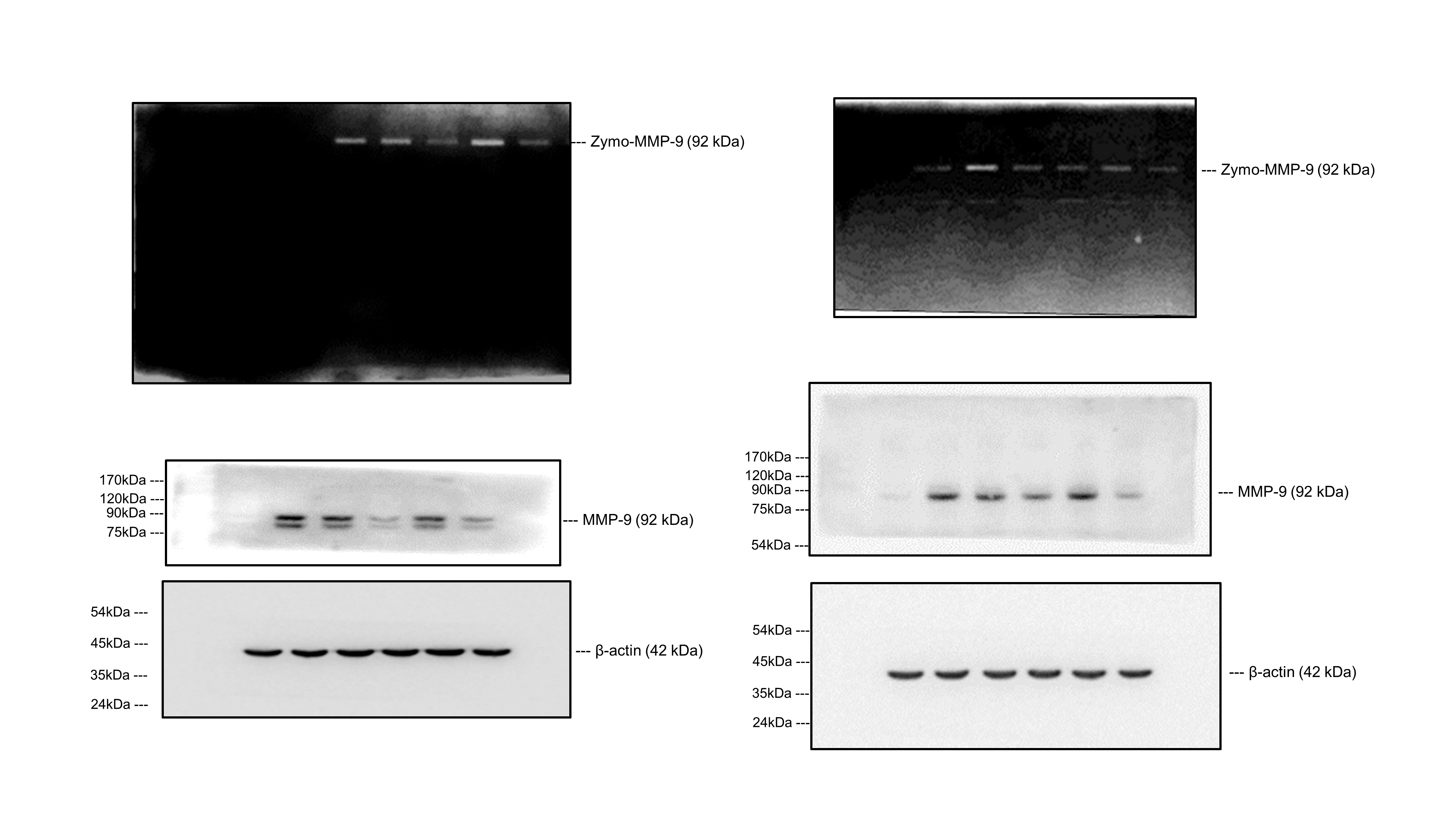

Supplement: Supplementary file 9 — Supplementary Figure 2. [file 41598_2022_16405_MOESM9_ESM.png]
